# Downregulation of METTL7B Inhibits Proliferation of Human Clear Cell Renal Cancer Cells *In Vivo* and *In Vitro*

**DOI:** 10.3389/fonc.2021.634542

**Published:** 2021-02-26

**Authors:** Wei Li, Shi Xu, Naixiong Peng, Zejian Zhang, Hua He, Ruoyu Chen, Dong Chen, Jiqing Fan, Xisheng Wang

**Affiliations:** ^1^ Department of Urology, Shenzhen Longhua District Central Hospital, The Affiliated Central Hospital of Shenzhen Longhua District, Guangdong Medical University, Shenzhen, China; ^2^ Department of Burn and Plastic Surgery, Shenzhen Longhua District Central Hospital, The Affiliated Central Hospital of Shenzhen Longhua District, Guangdong Medical University, Shenzhen, China; ^3^ Department of Proctology, Shenzhen Longhua District Central Hospital, The Affiliated Central Hospital of Shenzhen Longhua District, Guangdong Medical University, Shenzhen, China

**Keywords:** METTL7B, methyltransferase, ccRCC, proliferation, tumorigenesis

## Abstract

Clear cell renal cell carcinoma (ccRCC) is the most aggressive urologic tumor, and its incidence and diagonosis have been continuously increasing. Identifying novel molecular biomarker for inhibiting the progression of ccRCC will facilitate developing new treatment strategies. Although methyltransferase-like 7B (METTL7B) was identified as a Golgi-associated methyltransferase, the function and mechanism of METTL7B in ccRCC development and progression has not been explored. METTL7B expression were significantly upregulated in ccRCC tissues (n = 60), which significantly associated with TNM classification, tumor size, lymph node metastasis, and poor prognosis for ccRCC patients. Functional studies showed downregulation of METTL7B inhibited cell proliferation, migration *in vitro*, and xenograft tumor formation *in vivo*. In addition, METTL7B knockdown promoted cell cycle arrest at G0/G1phase and induced cellular apoptosis. Taken together, downregulation of METTL7B inhibits ccRCC cell proliferation and tumorigenesis *in vivo* and *in vitro*. These findings provide a rationale for using METTL7B as a potential therapeutic target in ccRCC patients.

## Introduction

Renal cell carcinoma (RCC) is a malignant tumor originated from the proximal tubules of the nephrons (1). Clear cell RCC (ccRCC) is the most prevalent pathological subtype and accounts for approximately 70% of all diagnosed RCC cases (2). Approximately 15–20% of ccRCC patients had metastatic lesions at primary diagnosis, and 30% eventually developed metastatic after surgery (1, 3). Once metastasized, the prognosis for patients is poor. The most critical causes of the poor outcome are the lack of sensitive biomarkers for early diagnosis and effective treatments (4). Therefore,it is needed to identify novel molecular targets, especially previously unrecognied molecules that can monitor the progression of ccRCC and develop more effective treatment strategies.

Methyltransferases (METTL) are a large group of enzymes that transfer a methyl group to lysine or arginine side chains on nuclear and cytoplasmic proteins (5). So the funtions of this protein family were predicted to methylate RNA (METTL3, METTL14, METTL1), DNA (METTL4), or protein (METTL10 and METTL11A) (6–8). Many studies have demonstrated that METTL proteins play a central role in the development of genetic diseases, cancers, and metabolic diseases through regulating signaling pathways (9, 10). For example, METTL14 suppresses proliferation and metastasis of colorectal cancer by downregulating oncogenic long non-coding RNA XIST (11). Upregulated METTL3 promotes metastasis of colorectal cancer *via* miR-1246/SPRED2/MAPK signaling pathway (12). Howerver, most METTL proteins are still poorly characterized.

METTL7B was originally identified as a Golgi-associated methyltransferase which methylates Golgi-associated proteins using multidimensional protein identification technology in 2004 (13). However, few study has investigated the function and mechanism of METTL7B in cancer development and progression. METTL7B might serve as a biomarker for diagnosis and tumor progression in papillary thyroid carcinoma (14). METTL7B also enhanced migration and invasion of thyroid carcinoma cells through promote TGF-β1-induced epithelial-mesenchymal transition (EMT) (15). Our previous study also showed that METTL7B is required for cancer cell proliferation and tumorigenesis in non-small cell lung cancer (16). Therefore, we planned to characterize the role and mechanism of METTL7B in ccRCC. In this study, we showed that METTL7B is involved in the regulation of cell cycle progression and essential for ccRCC development. We suggested that METTL7B might serve as a potential therapeutic target for ccRCC.

## Materials and Methods

### Clinical Samples and Immunohistochemistry (IHC) Staining

The tissue array containing a total of 60 pairs of ccRCC samples and matched adjacent normal renal tissues with follow-up data was obtained from Shenzhen Longhua District Central Hospital (Shenzhen, China). This study was approved by the Ethics Committee of Shenzhen Longhua District Central Hospital. IHC assays were performed on tissue microarray chips according to a standard protocols by Abclonal description. Briefly, sections were incubated with anti-METTL7B polyclonal antibody (Abclonal, #A7200, 1:100) overnight at 4°C, and subsequently incubated with streptavidin-conjugated horseradish peroxidase. Sections were visualized with DAB kit and counterstained with hematoxylin, mounted in neutral gum, and analyzed using a brightfield microscope. All IHC samples were assessed by two independent pathologists blinded to both the sample origins and the subject outcomes. For survival analyses, patient overall survivals stratified by METTL7B expression, were presented as the Kaplan–Meier plots and tested for significance using log-rank tests. Differences were considered significant when P value was less than 0.05.

### Cell Lines and Cultures

The human ccRCC cell line (786-O, A498, Caki-1,796-P, and ACHN) were purchased from the American Type Culture Collection (ATCC). HK 2 cell line was purchased from Shanghai Cell Bank, Chinese Academy of Sciences. The three ccRCC cell line(A498, Caki-1, and ACHN)were cultured in in Eagle’s Minimum Essential Medium (DMEM, Gibco) medium. The 786-O and 796-P were cultured in RPMI 1640 (Gibco). The normal proximal tubule epithelial cell line HK-2 was DMEM/F-12 1:1 (Gibco). All the cells were cultured in 10% fetal bovine serum (Hyclone) at 37°C in an incubator with 5% CO_2_. All lines were measured to be confirm negative of mycoplasma contamination.

### Cell Transfection

Cells were seeded on six-well plates at a density of 2 ×10^5^ cells/well. After 80% confluence was reached, shMETTL7B (METTL7B shRNA: 5’- GGGAAAGGCTGTCAAATAA -3’) and a negative control shRNA (shNC: 5’- TTCTCCGAACGTGTCACGT -3’) were transfected into cells using SuperFectin shRNA Transfection Reagent (Pufei, Shanghai, China). After 48 h, quantitative RT-PCR and Western blot were performed to determine the transfection efficiency.

### Cell Viability and Proliferation Assay

The living cell population was analyzed using Trypan Blue dye exclusion assay and MTT assay according to our previous report (17).

### Colony Formation Assay

METTL7B shRNA or negative control shRNA was transfected into 796-P and ACHN cells. Subsequently, cells were seeded in six-well plates at a density of 10^3^ cells/well. After incubation at 37°C for 14 days, colonies were fixed with 4% paraformaldehyde and stained by crystal violet (0.1%) for 15 min at room temperature and photographed by a camera. Macroscopic colonies of each well were counted.

### Flow Cytometry Analysis of Apoptosis and Cell Cycle Distribution

METTL7B shRNA or negative control shRNA was transfected into 796-P and ACHN cells. For apoptosis assays, cells were collected and treated with 500 μl Annexin V/PI binding buffer and then incubated for 15 min at room temperature. For cell cycle assays, cells were collected and fixed in 75% ethanol for 24 h. Then the cells were washed by cold PBS, stained with Annexin V- FITC and PI for 20 min, and then analyzed by flow cytometer (BD Bioscineces, Bedford, MA, USA).

### Cell Migration and Invasion Assays

The transwell insert for 24-well plate (8 μm-pore size, Corning, NY, USA) was used to measure the migratory and invasive ability of cells. For transwell migration assays, cells (2.5 × 10^4^) with shMETTL7B or negative control shRNA were seeded into the transwell insert with serum-free medium and the culture medium with 10% FBS was added in the lower chamber for chemo-attractant. The invasion assays were performed similarly except that the upper chambers of 24-well cell culture inserts were coated with 200 mg/ml of Matrigel (BD Biosciences, Bedford, MA). Following culture for 48 h at 37˚C, the cells in the bottom chamber were stained with 0.1% crystal violet (Beyotime Biotech, China). The cells were then evaluated by a light microscopy (CK40; Olympus Corporation, Tokyo, Japan) at the magnification at ×100. Images were captured, then the cells were counted randomly in five fields and the average was calculated.

### RNA Extraction and Real-Time Quantitative PCR Assays

Total RNA was extracted from cells using Trizol Reagent (Invitrogen, USA), and cDNA was synthesized from 1 μg of RNA with the M-MLV Reverse Transcriptase Kit (Promega, USA) as recommended by the manufacturer. Real-time quantitative PCR reactions for the quantification of gene expression were performed with Bio-Rad iQ5 Real Time PCR System. The primers for METTL7B and β-acin sequences used according to our previous report (16).

### Western Blot

Total protein was extracted as described previously (16, 18). Protein concentration was determined with the BCA Protein Assay Kit (Pierce, Rockford, IL, USA). Equivalent amounts of protein samples were uploaded and separated by 12% SDS-PAGE and then electro-transferred to polyvinylidene difluouride (PVDF) membranes (Millipore Corp, Atlanta, GA, USA). The membranes were blocked in 5% non-fat dry milk powder at room temperature for 1 h, and then incubated with primary antibodies as following: anti‐METTL7B (Abclonal, 1:800), anti‐N-cadherin (Abcam, 1:1,000), anti-vimentin (Abcam, 1:1,000), anti-slug (abcam, 1:1,000), anti-snail (CST, 1:800), anti-twist (CST, 1:800), anti-CDK1 (CST, 1:1,000), anti-CCND1 (CST, 1:1,000), anti-CCNB1 (CST,1:1,000), anti-CDKN2D (CST, 1:1,000) for overnight at 4°C, followed by HRP-conjugated secondary antibodies at room temperature for 1 h. The housekeeper gene β-actin was employed as an internal control. The signals of bands were detected by ECL reagents.

### *In Vivo* Tumorigenesis Assay

Male BALB/c nu/nu mice (4–5 weeks old) purchased from the Laboratory Animal Center of Shanghai, Academy of Science Chinese (Shanghai, China), were housed under specific pathogen-free conditions. Mice were randomly divided into two groups with five mice in each group. 796-P transfected with METTL7B shRNA or negative control shRNA (1 × 10^6^ cells/mice) were injected subcutaneously into the flanks of mice. Ten days after cell injection, the length (L) and width (W) of tumor xenografs were measured at a 3-day intervals with a vernier caliper. Tumor volumes were calculated (V = W^2^ × L/2). The animals were sacrificed under general anesthesia with chloral hydrate (5%, 100 μl/10 g). All the experiments complied with the guidelines of the Guangdong Medical University Institutional Animal Care and Use Committee on Animal Care and Use.

### Statistical Analysis

All data were expressed as mean ± standard deviation (S.D). SPSS 19.0 (SPSS, Chicago, IL, USA) was used to conduct all the statistical analysis. Kaplan-Meier for survival analysis. Student’s t-test were used to evaluate the differences between variables. Chi-square test or Spearman’s rank test (as appropriate) were used for correlation between METTL7B expression and clinicopathological characteristics. The correlation between gene expression and clinicopathologic features were and *P* value less than 0.05 was regarded as statistically significant.

## Results

### METTL7B Is Upregulated in ccRCC and Correlates With Poor Clinical Outcomes

**Figure 1:**
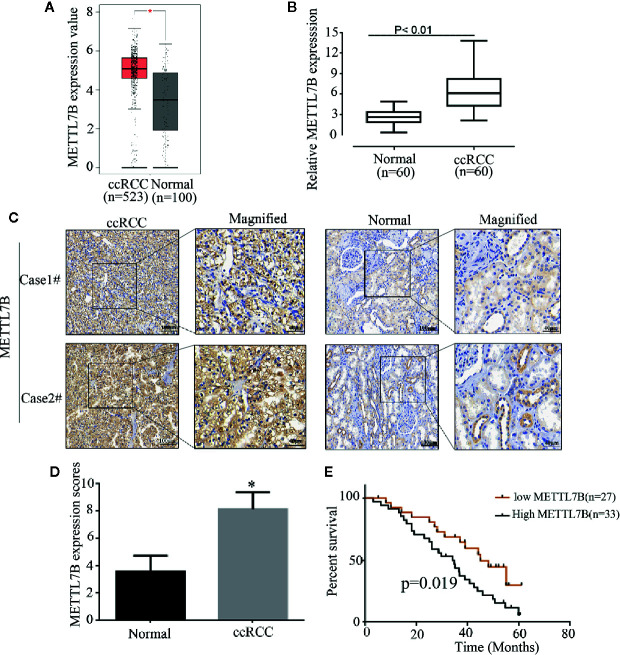
METTL7B is highly expressed in human ccRCC tissues. **(A)** The expressions of METTL7B in ccRCC tissues and normal renal tissues were analyzed based on TCGA database. Data are presented as the mean ± SD. **(B)** The expression of METTL7B mRNA were evaluated in 60 pairs of ccRCC tissues and normal renal tissues by qRT-PCR. Data are presented as the mean ± SD of three independent experiments. **(C)** The expressions of METTL7B in ccRCC and paired normal renal tissues were tested by IHC staining. **(D)** METTL7B staining scores in ccRCC tumors and the corresponding non-tumor tissues (n = 60). **(E)** Kaplan–Meier survival curves showed that METTL7B expression level was negatively correlated with prognosis prediction of ccRCC analyzed. *p < 0.05.

Firstly, to analyze the expression pattern of METTL7B in ccRCC, we compared METTL7B expression between normal renal tissues and ccRCC tissues using TCGA data. The results showed that METTL7B was significantly upregulated in ccRCC tissues (**Figure 1A**). To further investigate the clinical significance of METTL7B expression in RCC tumorigenesis, the expression of METTL7B was further evaluated in 60 pairs of ccRCC tissues and their matched non-tumor tissues by qRT-PCR and IHC staining. The results showed that the expression of METTL7B was significantly higher in ccRCC tissues compared to their matched normal renal tissues (**Figure 1B**). IHC staining analysis also indicated that the high expression of METTL7B in ccRCC tissues (**Figures 1C, D**).

The correlation of METTL7B expression with various clinicopathologic features was investigated and the result showed that upregulation of METTL7B was significantly associated with TNM classification (P = 0.048), Tumor size (P = 0.031), Lymph node metastasis (P = 0.046) (**Table 1**). Furthermore, the Kaplan-Meier survival analysis revealed that the patients harboring higher METTL7B expression showed significantly shorter overall survival than did patients with lower METTL7B expression (P < 0.019, **Figure 1E**). Taken together, the initial findings indicate that upregulation of METTL7B may play an important role in ccRCC tumorigenesis.

**Table 1:** The correlation between METTL7B expression and clinical characteristics of patients with ccRCC.

Characteristics	Cases(n = 60)	METTL7B Expression		*P value*
		Low	High	
Gender				
Male	26	11	15	0.714
Female	34	16	18	
Age				
>60 years	33	16	17	0.236
≤60 years	27	9	18	
Fuhrman Grade				
1–2	31	12	19	0.455
3–4	29	14	15	
TNM classification				
I-II	25	15	10	0.048^*^
III-IV	35	12	23	
Tumor size				
≤5cm	34	20	14	0.031^*^
>5cm	26	8	18	
Lymph node metastasis				
Negative	15	9	6	0.046^*^
Positive	45	14	31	

^*^means statistically significant. The meaning of the bold values is highlighted and less than 0.05.

### Downregulation of METTL7B Inhibits ccRCC Cell Proliferation *In Vitro*

To further investigate the role of METTL7B in ccRCC progression, we examined the expression of METTL7B in five ccRCC cell lines: 786-O, 796-P, A498, ACHN and Caki-1, and human normal proximal tubule epithelial cell line HK-2. Consistent with the result of ccRCC tissues, we found that METTL7B was upregulated in RCC cells compared with human normal proximal tubule epithelial cell HK-2 (**Supplementary Figure 1**). Among these ccRCC cells, 796-P and ACHN cell was predominantly upregulated. Therefore, we used 796-P and ACHN cells for further investigation in the following studies.

**Figure 2:**
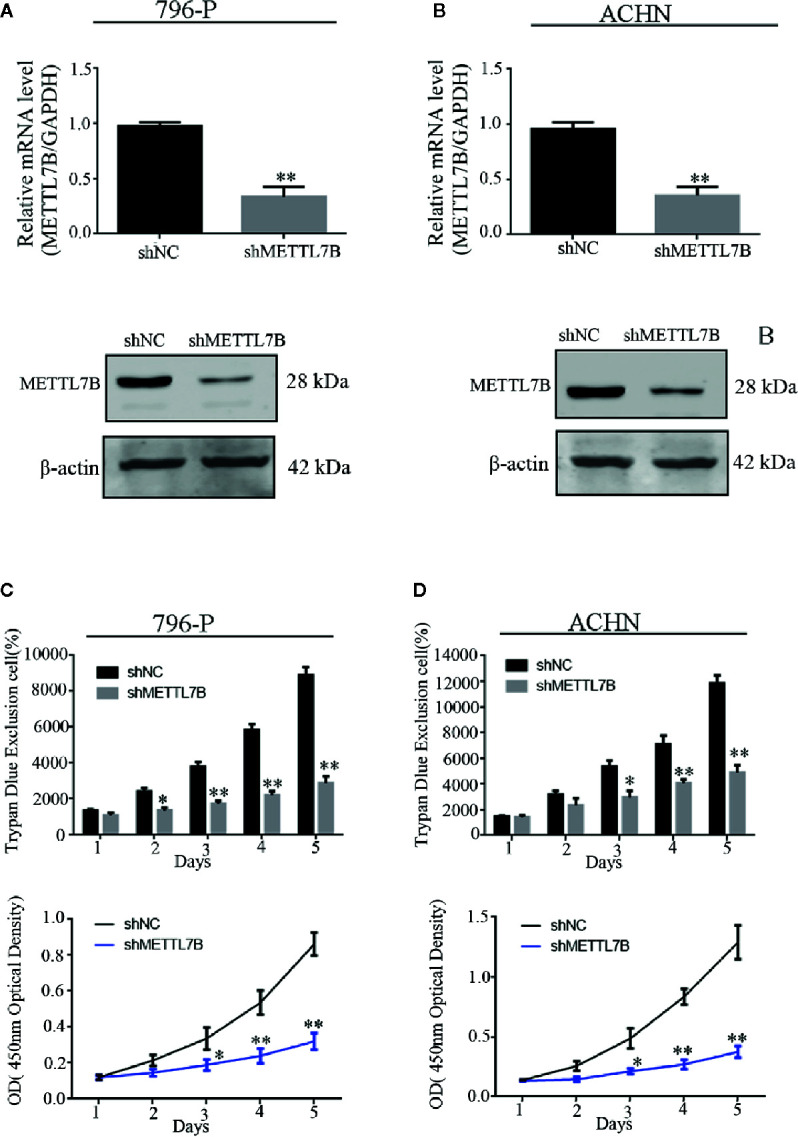
Knockdown of METTL7B inhibits the proliferation of ccRCC cells *in vitro*. 796-p and ACHN cells transfected with sh-METTL7B (shMETTL7B) or sh-Control (shNC) for 48 h. The transfection efficiency confirmed by qRT-PCR **(A)** and Western blot **(B)**. **(C)** The living cell population was analyzed using Trypan Blue dye exclusion assay. **(D)** the cell viability were measured using MTT assay. Data are presented as the mean ± SD of three independent experiments. **p < 0.01, *p < 0.05 *vs.* shNC group.

Our previous study have designed and validated two different shRNAs targeting METTL7B (16). One of them was used in this study. Firstly, 786-P and ACHN cells were transfected with lentivirus carrying a specific shRNA targeting METTL7B (shMETTL7B) and control (shCtrl). Lentivirus for knockdown efficiency was also examined by qPCR and western blot. The results showed that the mRNA and protein expressions of METTL7B were significantly decreased compared to that in the control group (shCtrl) (**Figures 2A, B**). Furthermore, the trypan blue rejection method and MTT assay were used to measure the cell proliferation and viability. Cell growth assay showed that METTL7B knockdown can significantly inhibit proliferation capabilities and viability (**Figures 2C, D**).

### Downregulation of METTL7B Induces ccRCC Cell Cycle Arrest At G1/S Transition

**Figure 3:**
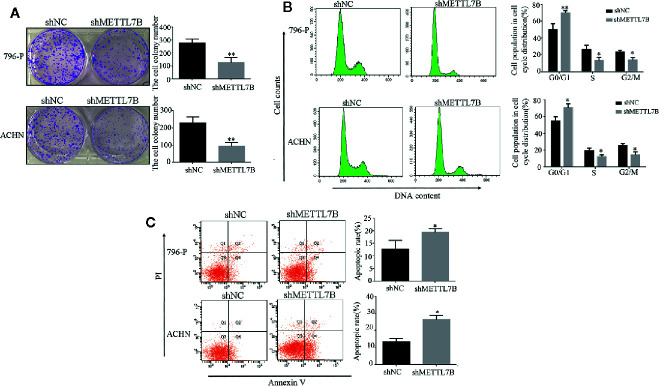
Knockdown of METTL7B promotes ccRCC cell cycle arrest and cellular apoptosis. **(A)** Colony-formation assay of 796-p and ACHN cells transfected with sh-METTL7B (shMETTL7B) or sh-Control (shNC). After incubation for 14 days, colonies were stained and photographed. *P < 0.05, *vs.* shNC group. **(B, C)** 796-p and ACHN cells transfected with sh-METTL7B (shMETTL7B) or sh-Control (shNC) were collected and analyzed using flow cytometry for cell apoptosis and cell cycle. Data are presented as the mean ± SD of three independent experiments. **p < 0.01, *p < 0.05 *vs.* shNC group.

Furthermore, we subsequently performed the colony formation assay to determine whether knocking down METTL7B could inhibit the colony formation capacity. Results demonstrated that knocking down METTL7B significantly reduced the number of colonies when compared with control cells (**Figure 3A**). Then we characterized the cell cycle transition and cellular apoptosis by flow cytometry analysis, and found that knocking down METTL7B increased the percentage of cells in G0/G1 phase from 55.51, 50.23% in shNC (796-P and ACHN) cells to 73.23, 69.18%, respectively in shMETTL7B cells (796-P and ACHN) (**Figure 3B**). In addition, based on the AnnexinV-FITC/PI staining assay, the percentages of apoptotic cells in shMETTL7B cells were significantly higher than that in shNC cells (**Figure 3C**). These data indicated that METTL7B is important for both ccRCC cell cycle transition and apoptotic signaling.

**Figure 4:**
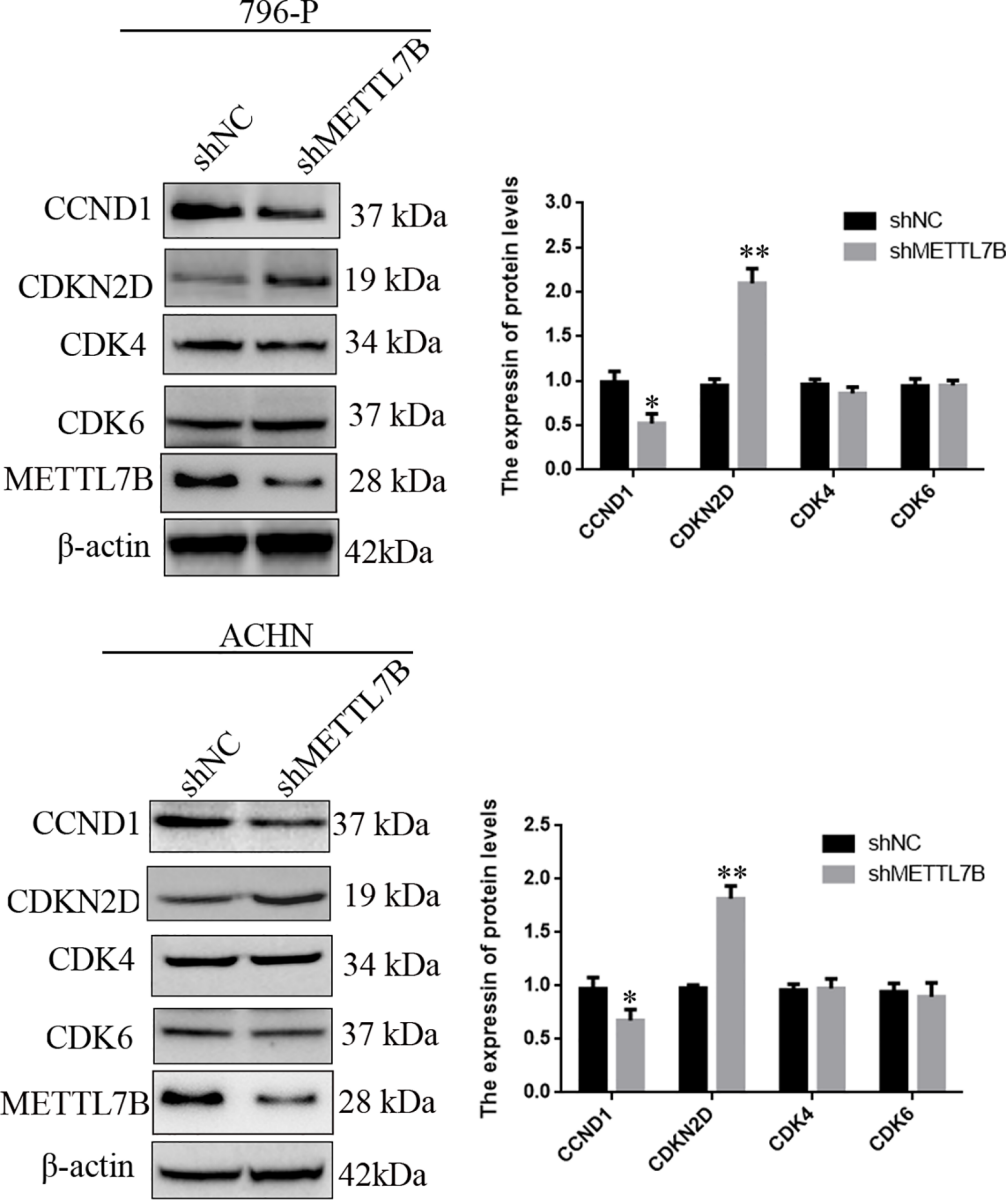
Knockdown of METTL7B inhibited G0/G1 realted protein expression. 796-p and ACHN cells transfected with sh-METTL7B (shMETTL7B) or sh-Control (shNC) for 48 h. The total protein was extracted and subjected to SDS-PAGE, followed by western blot analysis. Beta-actin was used as an internal control. Data are presented as means ± SD of three independent experiments. *P < 0.05, **P < 0.01 *vs* shCtrl group.

To investigate how METTL7B regulates cell cycle, the expression of G1/S transition regulators were measured by Western blot. The results showed that knocking down METTL7B significantly decreased the expression of CCND1 (Cyclin D1), but increased CDKN2D(cyclin dependent kinase inhibitor 2D) (**Figure 4**).

### Downregulation of METTL7B Inhibits ccRCC Cell Migration and Invasion

**Figure 5:**
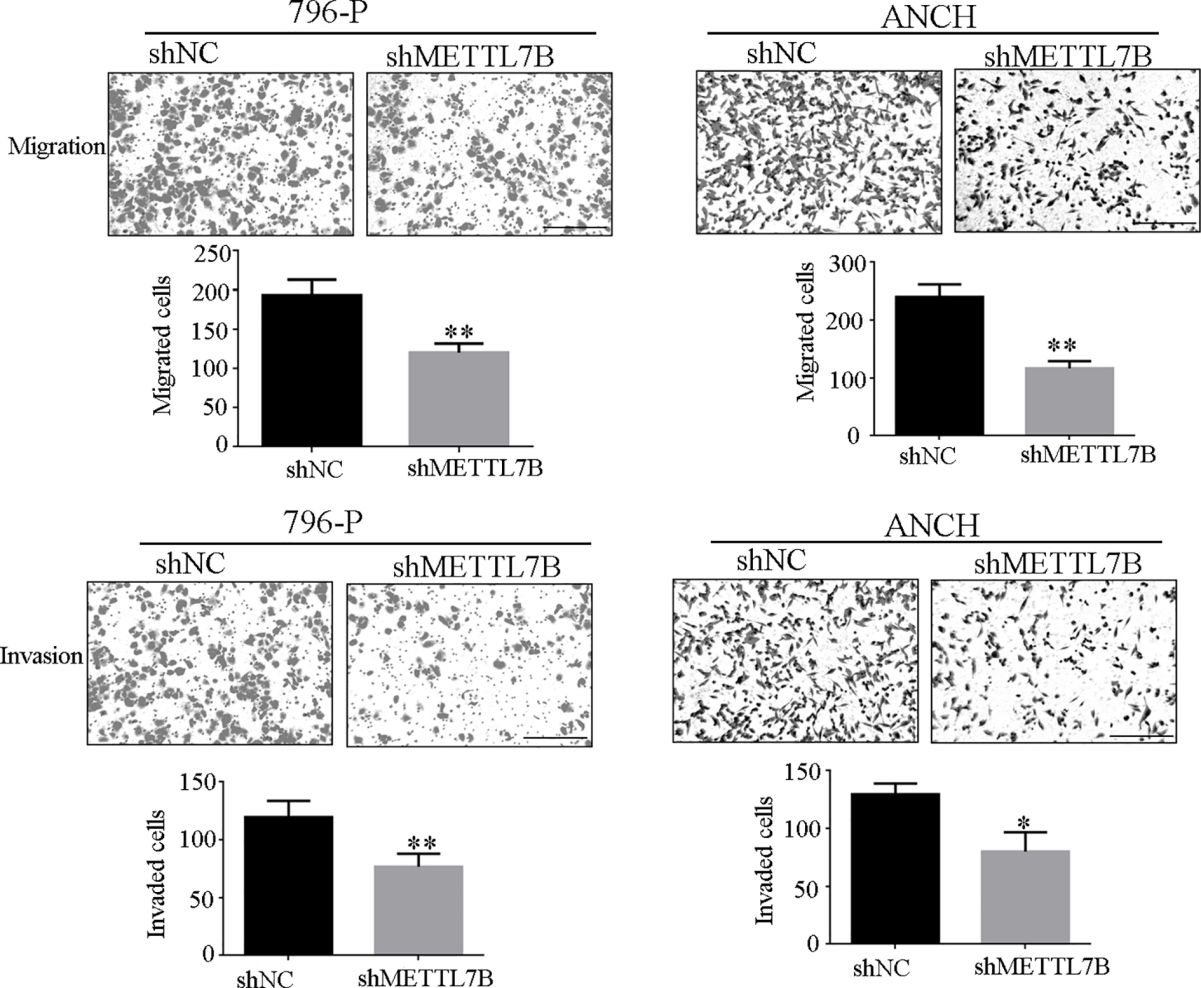
Knockdown of METTL7B inhibits ccRCC invasion and migration. 796-p and ACHN cells transfected with sh-METTL7B (shMETTL7B) or sh-Control (shNC) for 48 h. Invasion and migration assays were measured using Transwell champers. **(A)** Knockdown of METTL7B could inhibit cell migration in 796-p and ACHN cells. **(B)** Knockdown of METTL7B could inhibit cell invasion in 796-p and ACHN cells. Data represent mean ± S.D. of three independent experiments. Scale bar = 100 μm, *P < 0.05, **P < 0.01 *vs* shCtrl group.

**Figure 6:**
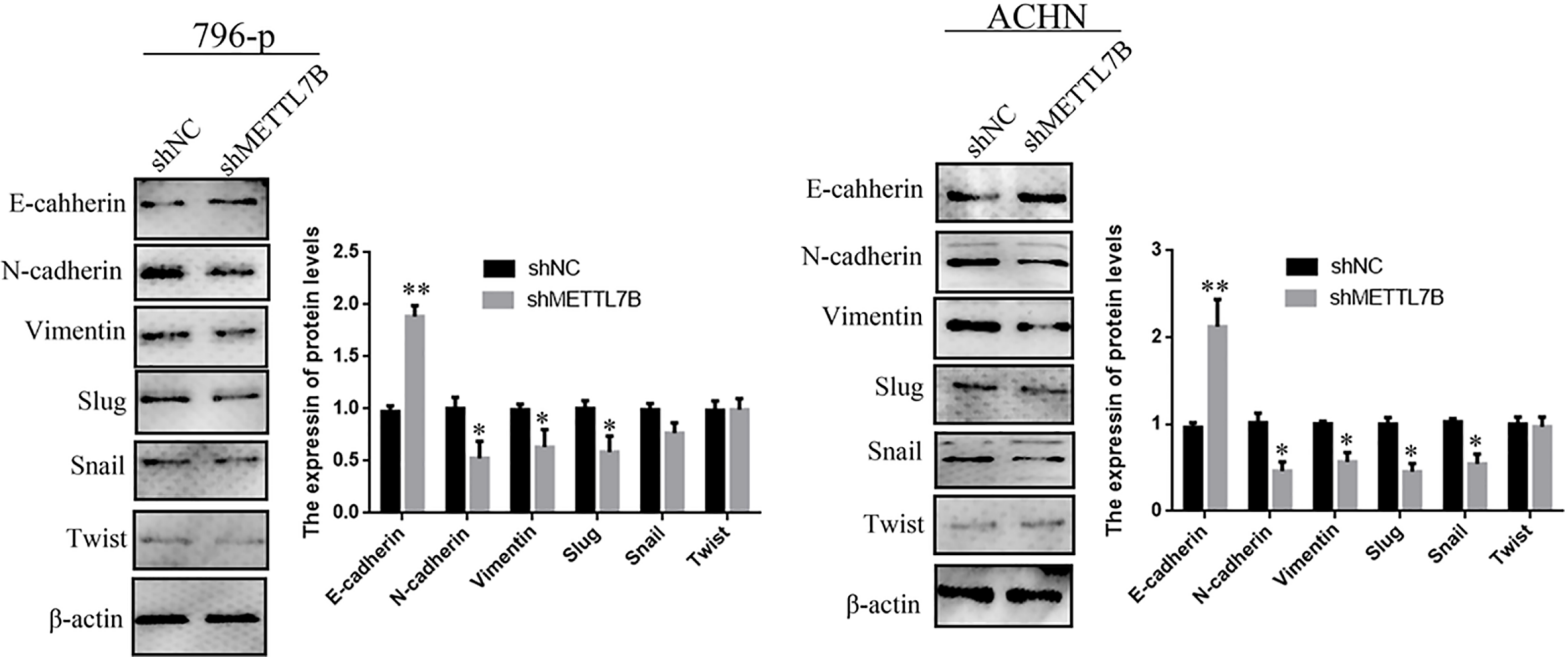
Knockdown of METTL7B inhibited EMT in ccRCC cells. A significant reduction of vimentin and N-cadherin but increase E-cadherin was detected in shMETTL7B cells compared to shNC cells by Western blotting. Beta-actin was used as an internal control. Data are presented as means ± SD of three independent experiments. *P < 0.05, **P < 0.01 *vs* shCtrl group.

Because METTL7B expression levels correlated with lymph node metastasis, the effect of METTL7B on ccRCC metastasis was also investigated. Transwell migration and invasion assays showed that knocking down METTL7B could significantly decrease cell motility and invasion (**Figure 5**). EMT was shown to strongly enhance cancer cell motility and metastasis, so the expression of several EMT-associated proteins were measured by Western blot. The results showed that knocking down METTL7B could significantly increase the expression of the epithelial markers E-cadherin, and inhibit the expression of mesenchymal markers N-cadherin, Vimentin, and Slug without affecting Snail and Twist expression in 796-P cells (**Figure 6**). In ACHN cells, knocking down METTL7B also inhibits Snail expression (**Figure 6**). These data indicated that knocking down METTL7B can inhibit ccRCC cell migration and invasion by inhibiting EMT.

### Downregulation of METTL7B Represses ccRCC Tumor Growth *In Vivo*

**Figure 7:**
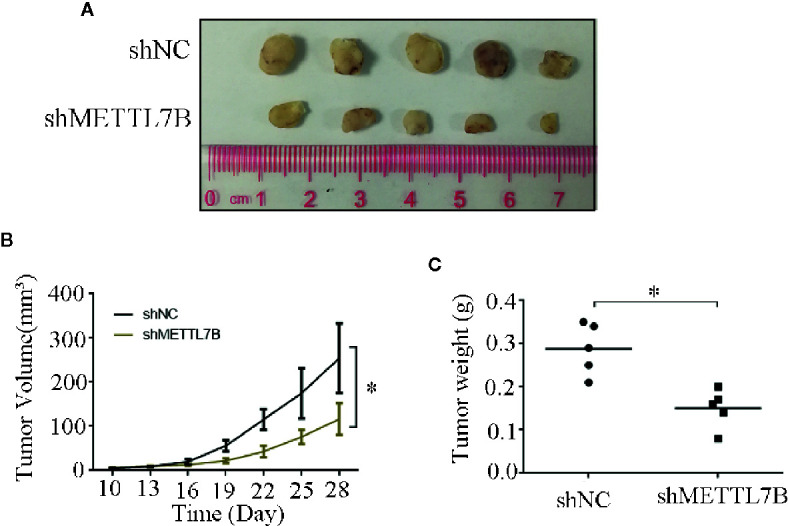
Knockdown of METTL7B inhibits xenograft tumor formation *in vivo*. **(A)** Representative xenograft tumors for indicated cells were shown. **(B)** METTL7B knockdown significantly reduced xenograft tumor growth in male nude mice by tumor volume examination. **(C)** Depletion of METTL7B significantly suppressed xenograft tumor weights. *P < 0.05 *vs* shCtrl group.

**Figure 8:**
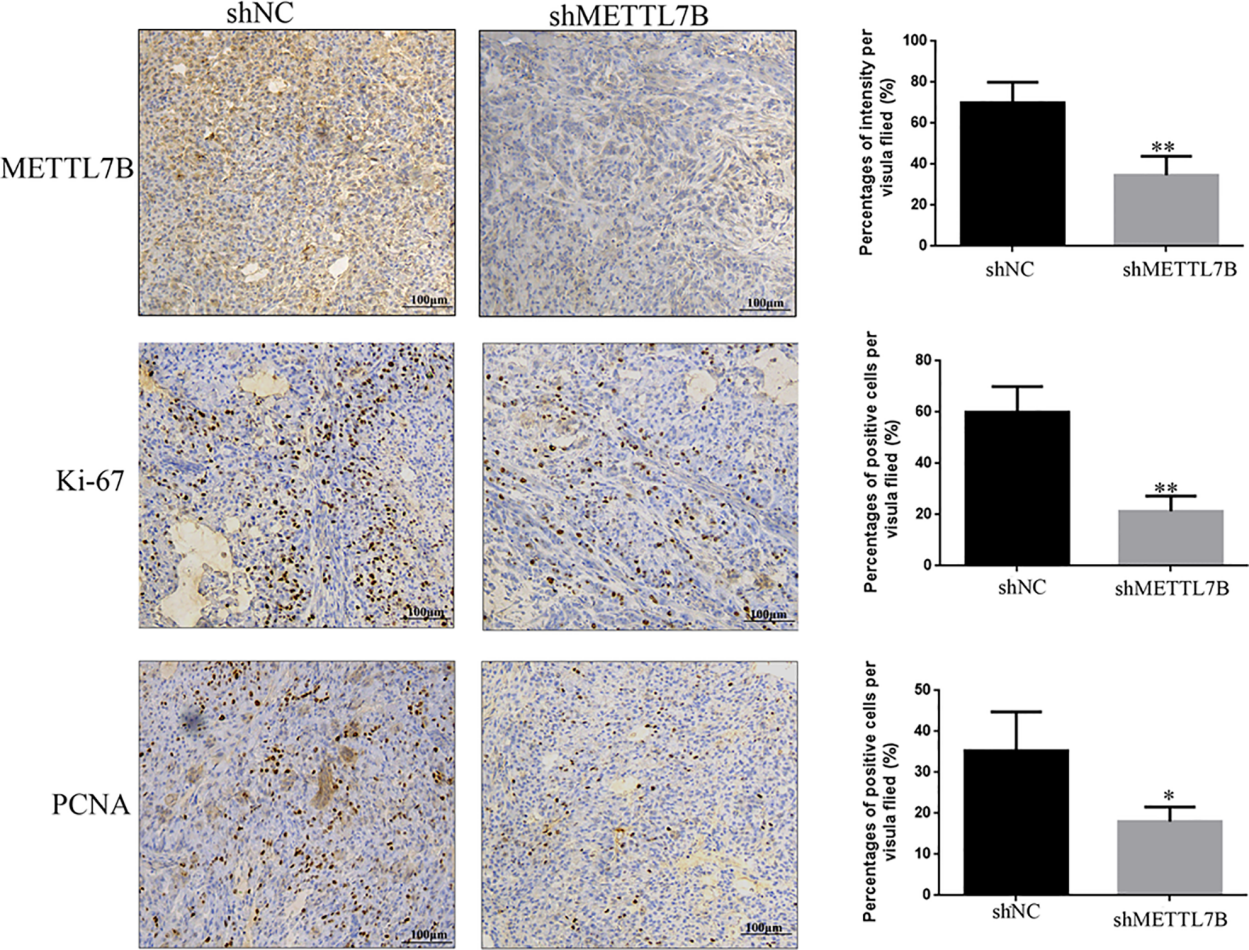
Knockdown of METTL7B inhibits the expression of Ki67 and PNCA in xenograft tumor tiusses. IHC staining were used to measured the expression of Ki67 and PNCA in xenograft tumor tiusses. The intensities of Ki-67 and PCNA were both decreased in xenograft tumors from shMETTL7B-transfected 796-p cells. *P < 0.05 vs shCtrl group.

To further study the biological functions of METTL7B *in vivo*, shMETTL7B-796-P and shNC-796-P cells were subcutaneously inoculated into BALB/c nude mice and tumor growth was monitored. The results showed that knocking down METTL7B significantly inhibited tumor growth compared with shNC group (**Figure 7A**). The tumor volume and weight from knockdown METTL7B tumor group was significantly smaller than shNC group (**Figures 7B, C**). However, there was no significant loss in body weight in the experimental mice. Taken together, these findings indicated that knocking down METTL7B can suppress ccRCC cells growth *in vivo.* Expression of Ki-67 antigen and proliferating cell nuclear antigen (PCNA) was assessed immunohistochemically in specimens from xenografts. The results showed that the expression level of Ki-67 and PCNA in shMETTL7B exnograft tumors was dramatically decreased (**Figure 8**). These findings demonstrated that METTL7B knockdown inhibited the growth of ccRCC *in vivo*.

## Discussion

METTL7B was firstly identified as a Golgi-associated methyltransferase (13). However, there was no report on the function of METTL7B in ccRCC cancer development. In the present study, we found that METTL7B is frequently upregulated in ccRCC tissues, which significantly associated with TNM classification, Tumor size, Lymph node metastasis, and poor prognosis for ccRCC patients. We further analyzed the role of METTL7B in ccRCC cell proliferation, tumorigenesis *in vitro* and *in vivo*, and explored the molecular mechanisms.

In the past, studies on molecular mechanisms of ccRCC tumorigenesis mainly focused on the oncogenes or tumor suppressor genes that coded proteins (2, 19). However, aberrant methylation may be the most common mechanism inactivating cancer-related genes in ccRCC. Previous studies have demonstrated that methylation links to cancer development as methylation of tumor suppressor genes promotes tumorigenesis (20, 21). In this study, we identified that both mRNA and protein of METTL7B is upregulated ccRCC tissues. Furthermore, overexpression of METTL7B was significantly associated with TNM classification, Tumor size, Lymph node metastasis, and poor prognosis for ccRCC patients. Previous studies have showed that m6A related genes (METTL3, METTL14, and HNRNPA2B1) may predict the prognosis of ccRCC patients (22). This results indicated that METTL7B might be a potential prognostic biomarker in ccRCC in the further.

We analyzed the function of METTL7B in the growth of ccRCC cells. The results showed that knockdown METTL7B significantly inhibited cell proliferation, clone formation. Furthermore, knockdown METTL7B promotes cell cycle arrest at G0/G1 phase and cellular apoptosis. These results suggested that METTL7B affect cell cycle progression through inhibiting or increasing cell cycle related-genes expression. We examined the expression of G0/G1 related genes using Western blot. The results showed that the expression of CCND1 were significantly downregulated in shMETTL7B cells, while cyclin dependent kinase inhibitor 2D (CDKN2D) was significantly upregulated without affecting CDK4 and CDK6 expression. These results were consistent with cell cycle arrest in our study and previous reports. Previous studies have demonstrated that CCND1 can bind with CDK4 to control cell proliferation and migration (23). CCND1 and CDK4–mediated cell cycle progression provides a competitive advantage for human hematopoietic stem cells *in vivo* (24). CCND1 paly significant roles in the regulation of RCC cell proliferation and tumorigenesis. LINC00511 can promote the malignant phenotype of ccRCC by increasing CCND1 expression (25). Furthmore, high amounts of CDKN2D also can inhibit CDK4/6 activity and further arrest the cell cycle progression from G1 to S phase (26). miR-451 inhibits esophageal carcinoma proliferation by targeting CDKN2D expression (27). These results suggested that METTL7B may function as a oncogene and play a critical role in ccRCC formation and progression.

Epithelial-mesenchymal transition (EMT) plays an important role in both development, cancer progression, and metastasis (28). We further investigate the effect of METTL7B on ccRCC cell invasion. The results were consistent with previous report that knockdown METTL7B significantly inhibited cell invasion and migration (15). Loss of component molecules of cell adhesion and tight junctions is the hallmark of EMT in cancer (28, 29). We then examined changes in expression levels of key EMT-related transcription factors and markers in ccRCC cells after METTL7B knockdown. The results showed that knockdown METTL7B increased the expression of E-cadherin, but reduced the expression of N-cadherin, and vimentin. In addition, the expression of Slug (transcription factors) was significantly repressed in METTL7B knockdown cells, whereas the expression of Twist1, Snail, Zeb1, or Zeb2 was not changed in this context. However, mechanisms of how METTL7B up- or downregulates the expression of these genes needs to be further investigated.

Prvious studies have demonstrated that METTL proteins play a important role in tumorgenesis through methylate RNA, DNA or protein (6–8). In addition, Gene Ontology (GO) annotations showed METTL7B has methyltransferase activity and S-adenosylmethionine-dependent methyltransferase activity. So we think that METTL7B might affects protein, DNA or RNA methylation. In the following study, We will further study the molecular regulatory mechanism through whole genome methylation sequencing and RNA methylation sequencing to confirm that how METTL7b can regulate cell cycle or EMT-related gene expression.

In conclusions, our study demonstrated that METTL7B is aberrantly overexpressed in ccRCC tissues. Knocking down METTL7B can significantly reduce ccRCC cell proliferation both *in vivo* and *in vitro.* The oncogenic role of METTL7B is achieved by inhibiting the expression of genes involved in the regulation of cell-cycle (such as CDK1, CCND1, and CCNB1) and invasion (E-cadherin, N-cadherin, Vimentin, and Slug).

## Data Availabiltiy Statement

The original contributions presented in the study are included in the article/**Supplementary Material**. Further inquiries can be directed to the corresponding authors.

## Ethics Statement

This study was approved by the Ethics Committee of Shenzhen Longhua District Central Hospital. The patients/participants provided their written informed consent to participate in this study.

## Author Contributions

WL, SX, and XW developed the project. WL, RC, and NP performed experiments and wrote the manuscript. ZZ,HH, DC, and JF supervised the work. All authors contributed to the article and approved the submitted version.

## Funding

The work was supported by grants from the National Natural Science Foundation of China (No. 81702889).

## Conflict of Interest

The authors declare that the research was conducted in the absence of any commercial or financial relationships that could be construed as a potential conflict of interest.

## Supplementary Material

Supplementary Figure 1 Relative METTL7B expression in five ccRCC cell lines (786-O, A498, Caki-1, 796-P, and ACHN) and one normal human renal cell line (HK-2) was measured by RT-pPCR and Western blot, *P < 0.05 *vs* HK-2 group.

## Abbreviations

METTL7B, Methyltransferase 7B; RCC, Renal cell carcinoma; ccRCC, Clear cell RCC; PCR, Polymerase Chain Reaction; shRNA, Short hairpin RNA; BSA, Bovine Serum Albumin; h, hour; CCK8, Cell Counting Kit; S.D., standard deviation; EMT, Epithelial-mesenchymal transition; CDKN2D, cyclin dependent kinase inhibitor 2D; PCNA, proliferating cell nuclear antigen.
