# Supplementary figures and images for: Downregulation of METTL7B Inhibits Proliferation of Human Clear Cell Renal Cancer Cells In Vivo and In Vitro
Source: Front Oncol. 2021 Feb 26;11:634542. doi: 10.3389/fonc.2021.634542 (PMC7952878; doi:10.3389/fonc.2021.634542)

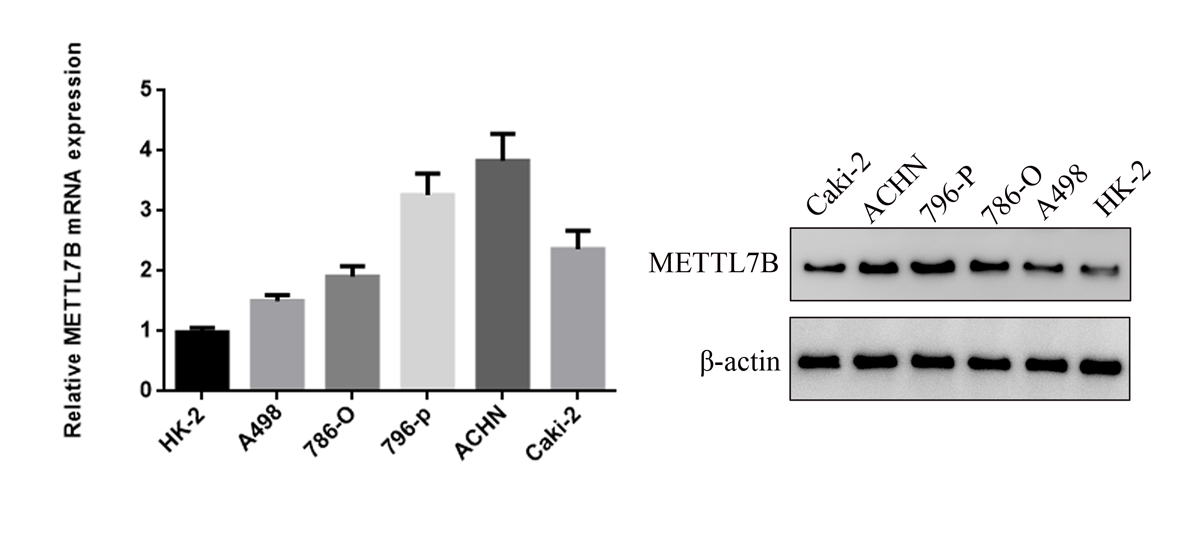

Supplement: Supplementary Figure 1 — Relative METTL7B expression in five ccRCC cell lines (786-O, A498, Caki-1, 796-P, and ACHN) and one normal human renal cell line (HK-2) was measured by RT-pPCR and Western blot, *P < 0.05 vs HK-2 group. [file Image_1.tif]
